# Atrial fibroblast–derived macrophage migration inhibitory factor promotes atrial macrophage accumulation in postoperative atrial fibrillation

**DOI:** 10.1172/jci.insight.190756

**Published:** 2025-08-14

**Authors:** Joshua A. Keefe, Jose Alberto Navarro-Garcia, Shuai Zhao, Mihail G. Chelu, Xander H.T. Wehrens

**Affiliations:** 1 Cardiovascular Research Institute,; 2 Department of Integrative Physiology, and; 3 Department of Medicine (Division of Cardiology), Baylor College of Medicine, Houston, Texas, USA.; 4 Texas Heart Institute at Baylor St. Luke’s Medical Center, Houston, Texas, USA.; 5 Department of Neuroscience,; 6 Department of Pediatrics (Division of Cardiology), and; 7 Center for Space Medicine, Baylor College of Medicine, Houston, Texas, USA.

**Keywords:** Cardiology, Immunology, Arrhythmias, Macrophages, Surgery

## Abstract

<br> New study: Blocking MIF protein prevents irregular heartbeats after surgery by reducing harmful immune cell buildup in the atria.

**To the Editor:** Macrophage migration inhibitory factor (MIF) is a pro-inflammatory cytokine with macrophage chemoattractant properties (1). Prior work implicated MIF in atrial fibrillation (AF) through fibrosis and calcium mishandling (2), but no studies have assessed whether MIF plays a role in postoperative AF (poAF) through its macrophage chemoattractant function (1). We previously demonstrated that pro-inflammatory atrial macrophages are increased in patients and mice with poAF and that triggered activity is a central arrhythmogenic mechanism in poAF downstream of postsurgical inflammation (3–5). Here, we explored whether MIF drives upstream atrial macrophage infiltration after cardiac surgery.

Using our published poAF mouse model, we conducted single-cell RNA sequencing (scRNA-Seq) comparing atrial nonmyocytes isolated on postoperative day three from mice with poAF (thoracotomy AF [TAF]) versus sham and found macrophages and neutrophils to be the most prominently altered cell types (4). To elucidate molecular pathways driving atrial macrophage infiltration, we conducted CellChat, which revealed MIF as a top communication pathway in TAF mice, particularly between atrial fibroblasts (AFB) and macrophages (Figure 1A). Reclustering of AFB and macrophages led to 4 AFB and 5 macrophage clusters (Figure 1B). Interestingly, *Mif* was prominently upregulated in alpha-2 actin–positive (*Acta2*^+^) myofibroblasts (Supplemental Figure 1, A and B; supplemental material available online with this article; https://doi.org/10.1172/jci.insight.190756DS1), in addition to being globally elevated in the atria in TAF versus sham mice (Figure 1C and Supplemental Figure 1C), consistent with the notion that *Mif* is ubiquitously expressed (6).

To directly test whether MIF was necessary for poAF, we performed cardiac or sham surgery in mice given vehicle or the MIF inhibitor, 4-IPP (Figure 1D) (7). Strikingly, 4-IPP prevented poAF (5.2-fold reduction, *P* = 0.036; Figure 1E) and reduced poAF duration (9.4-fold, *P* = 0.037; Supplemental Figure 2, A and B). Importantly, MIF inhibition attenuated atrial macrophage infiltration by Western blot (2.0-fold reduction, *P* = 0.024; Figure 1F) and flow cytometry (1.6-fold reduction, *P* = 0.048; Figure 1G) without changes in atrial neutrophil accumulation (Supplemental Figure 3, A and B), indicating that MIF is necessary for postsurgical atrial macrophage infiltration. Interestingly, MIF inhibition did not alter CXCR2 expression (Figure 1F), which may play a role in MIF-driven macrophage recruitment (1), suggesting that blocking MIF itself rather than its receptor may portend greater therapeutic utility. Together, MIF is necessary for atrial macrophage recruitment after cardiac surgery.

To assess the translatability of our findings, we collected pericardial fluid (PF) from patients 24–36 hours after cardiac surgery as a surrogate of local cardiac inflammation (4, 8). Strikingly, MIF was significantly greater in the PF of patients with poAF versus sinus rhythm (SR) (1.7-fold, *P* = 0.038) 24 hours after open-heart surgery (Figure 1H and Supplemental Table 1), suggesting that an early surge of MIF may recruit macrophages. To explore this finding, we challenged THP-1 monocytes in vitro with PF in the presence or absence of MIF inhibitor 4-IPP. Treatment of THP-1 monocytes with PF increased *IL-1b* expression (Supplemental Figure 4) and STAT3-Tyr705 phosphorylation (Figure 1I), both of which were attenuated by MIF inhibition. Interestingly, while *IL-1b* expression was greater in SR versus poAF PF (Supplemental Figure 4), STAT3-Tyr705 phosphorylation showed the opposite trend — that challenge with poAF PF led to significantly greater STAT3-Tyr705 phosphorylation compared with challenge with SR PF (Figure 1I), consistent with prior findings (4). Importantly, MIF inhibition attenuated IL-1β– and IL-6–mediated inflammation in PF-stimulated THP-1 monocytes, suggesting that MIF is a critical mediator of macrophage-driven inflammation.

Together, MIF is necessary for atrial macrophage recruitment and poAF. While activated AFB may be key drivers of MIF secretion, conditional knockout of *Mif* in these *Acta2*^+^ myofibroblasts is warranted. Taken together, our findings suggest that pericardial MIF may portend prognostic utility for poAF given its unique upregulation in patients with poAF prior to the onset of poAF, in contrast with other cytokines (8), as well as therapeutic utility given our murine findings.

All data in this study are available in the Supporting Data Values as well as from the corresponding author upon reasonable request. Data from scRNA-Seq experiments are publicly available in the National Center for Biotechnology Information BioProject Repository (PRJNA1220606).

## Supplementary Material

Supplemental data

Unedited blot and gel images

Supporting data values

## Figures and Tables

**Figure 1:**
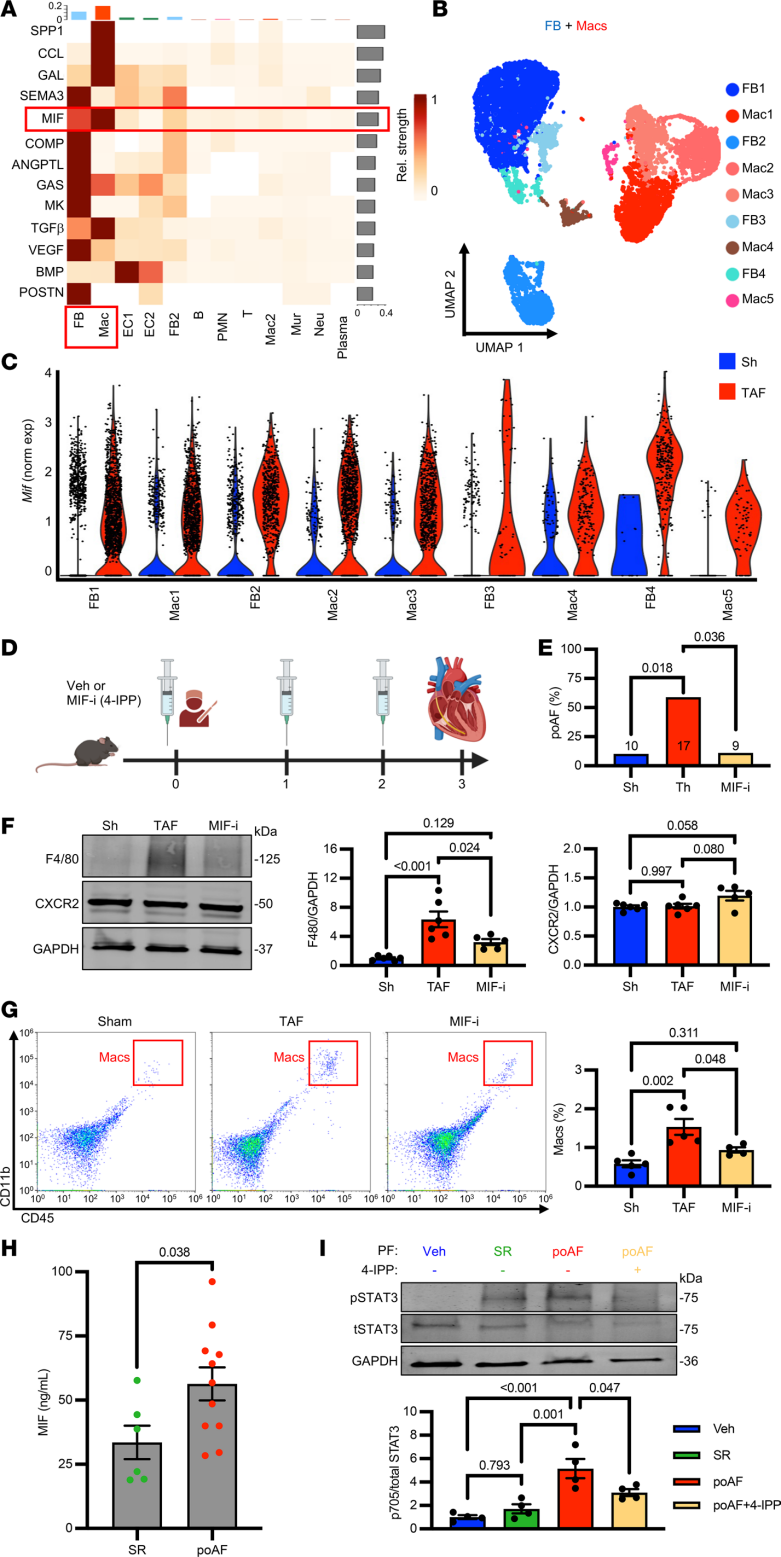
MIF drives atrial macrophage recruitment in poAF. (**A**) scRNA-Seq comparing atrial nonmyocytes from Sh (*n* = 3) versus TAF (*n* = 3) mice revealed MIF as a top communication pathway. (**B**) Reclustering of AFB and macrophages with (**C**) quantification of *Mif* expression. Data shown as mean ± IQR. (**D** and **E**) MIF inhibition with 4-IPP (50 mg/kg/d) prevented poAF in mice (χ^2^ test). Number of mice per group is denoted in **E**. Atrial macrophage accumulation by (**F**) F4/80 protein (*N* = 6 per group) and (**G**) flow cytometry (*N* = 4 per group) were attenuated by 4-IPP without changes in CXCR2 (*N* = 6 per group). *P* values were derived from 1-way ANOVA followed by Tukey’s. (**H**) MIF was elevated in PF from poAF (*N* = 11) versus SR (*N* = 6) patients 24 hours after cardiac surgery (2-sample 2-tailed *t* test). (**I**) Treatment of THP-1 monocytes (*N* = 4 per group) with PF (1:10 dilution) for 6 hours increased STAT3-Tyr705 phosphorylation, which was attenuated by 30 minutes of 100 μM 4-IPP pretreatment (1-way ANOVA followed by Tukey’s). AFB, atrial fibroblast; EC, endothelial cell; FB, fibroblast; Mac, macrophage; MIF-i, macrophage migration inhibitory factor inhibitor; norm exp, normalized expression; PF, pericardial fluid; poAF, postoperative atrial fibrillation; scRNA-Seq, single-cell RNA sequencing; Sh, sham; SR, sinus rhythm; TAF, thoracotomy atrial fibrillation; UMAP, uniform manifold approximation and projection.

## Version 1 08/14/2025

In-Press Preview

## Version 2 09/23/2025

Electronic publication
