# Supplementary material for: Atrial fibroblast–derived macrophage migration inhibitory factor promotes atrial macrophage accumulation in postoperative atrial fibrillation
Source: JCI Insight. 2025 Aug 14;10(18):e190756. doi: 10.1172/jci.insight.190756 (PMC12487863; doi:10.1172/jci.insight.190756)
Supplement: Supplemental data [file jciinsight-10-190756-s063.pdf]

SUPPLEMENTAL MATERIAL

Atrial Fibroblast-Derived Macrophage Migration Inhibitory Factor Promotes Atrial Macrophage Accumulation in Postoperative Atrial Fibrillation

Joshua A. Keefe[1,2], Jose Alberto-Navarro Garcia[1,2], Shuai Zhao[1,2], Mihail G. Chelu[1,3,4], Xander H. T. Wehrens[1,2,3,5,6,7]

[1]Cardiovascular Research Institute, [2]Department of Integrative Physiology, [3]Department of Medicine (Division of Cardiology), Baylor College of Medicine, Houston, TX 77030, USA.
[4]Texas Heart Institute at Baylor St. Luke's Medical Center, Houston, TX 77030, USA.
[5]Department of Neuroscience, [6]Department of Pediatrics (Division of Cardiology), [7]Center for Space Medicine, Baylor College of Medicine, Houston, TX 77030, USA.

Running title: MIF Recruits Macrophages in Postoperative AF

Correspondence to: Xander Wehrens, M.D., Ph.D., Cardiovascular Research Institute, Baylor College of Medicine, BCM335, One Baylor Plaza, Houston, TX 77030. Email: wehrens@bcm.edu; ORCID: 0000-0001-5044-672X

METHODS

Study approval: All animal studies were performed according to protocols approved by the Institutional Animal Care and Use Committee of Baylor College of Medicine (AN-9166) conforming to the Guide for the Care and Use of Laboratory Animals published by the US National Institutes of Health (Publication no. 85-23, revised 1996).

Sex as a biological variable: All mice included in this study were 12-15 week-of-age, C57BL/6J background purchased from the Jackson Laboratory (Bar Harbor, ME), and equal numbers of male and female mice were used when possible. Controls consisted of wild-type littermates. All studies and analyses were performed in a blinded manner when possible.

Mouse open heart surgery was performed as previously described (1). Briefly, mice were given preprocedural analgesia with extended-release buprenorphine (Wedgewood Connect, San Jose, CA) at 1.0 mg/kg at least one hour prior to surgery. Mice were intubated and ventilated (tidal volume 150 µL, respiratory rate of 175 breaths/minute). Anesthesia was maintained using 2.5% v/v isoflurane (11695067771, Covetrus, Houston, TX) in 100% oxygen, and body temperature was maintained at 37.0±0.5ºC by rectal thermometer (Rodent Surgical Monitor+, Indus Instruments, Webster, TX). The thoracic cavity was exposed through the 2nd intercostal space. Bi-atrial pericardiectomy and cross-clamping of the thoracic aorta for 20 seconds were performed. For the sham (Sh) procedure, endotracheal intubation and skin and pectoral muscle dissection were

performed, but the thoracic cavity was not entered. MIF inhibitor, 4-IPP, was administered intraperitoneally at 50 mg/kg once a day from the time of surgery through postoperative day two (2).

Programmed Electrical Stimulation (PES) was performed as previously described (1, 3). Mice were anesthetized using 2% v/v isoflurane/oxygen, and a 1.1F octopolar catheter (EPR-800, Millar, Houston, TX) was inserted into the right atrium and ventricle through the right jugular vein. The octopolar catheter leads were connected to an external stimulator (STG3008, MultiChannel Systems, Reutlingen, Germany), and the signals were acquired using IOX2.4 acquisition software (Emka Technologies, Sterling, VA). Proper catheter positioning was verified by electrogram waveforms and response to atrial pacing (3). All PES protocols were performed at 1.75% v/v isoflurane/oxygen and rectal temperature between 37.0±0.5°C. AF inducibility was determined via decremental pacing by performing a series of 2s bursts with a pulse width of 1 ms. The starting BCL was 40 ms and was decreased by 2 ms after each 2s burst until a BCL of 20 ms was reached. Burst pacing was performed in triplicate for each mouse, and AF was defined as the presence of an irregularly irregular rhythm without discernable P waves for at least 1 second on at least two out of three atrial burst pacing protocols (1).

Single-cell RNA sequencing (scRNAseq): Atrial non-myocytes were isolated after PES studies by mechanical and enzymatic digestion using 450 U/mL collagenase II (C2-28, Sigma-Aldrich, St. Louis, MO), 125 U/mL collagenase XI (C7657, Sigma-Aldrich, St. Louis, MO), 60 U/mL DNase (D45131VL, Sigma-Aldrich, St. Louis, MO), and 60 U/mL

hyaluronidase (5030-9954, Bio-Rad, Hercules, CA), dissolved in Hanks Balanced Salt Solution (HBSS) with 1.26 mM Ca^{2+} and 0.9 mM Mg^{2+}. Atrial tissue pieces were placed in prewarmed digestion buffer in a water bath at 37.0ºC and triturated every 10 minutes for 30 minutes. The digestion reaction was quenched using 10% FBS in HBSS without Ca^{2+} and Mg^{2+}. Cells were filtered through a 70-μm filter and spun at 340 x g for 7 minutes at 4ºC. Cells were resuspended in RBC lysis buffer for 5 minutes on ice and then spun at 340 x g for 7 minutes at 4 ºC. Cells were resuspended in 5% FBS in HBSS without Ca^{2+} and Mg^{2+}. Single cell suspensions were loaded onto a 10X Genomics Chromium Controller using 10X Single Cell RNA reagents v3.1 (4). DNA libraries were generated following the Chromium scRNA-seq v3.1 protocol. DNA libraries were sequenced using a Next Generation Sequencer NovaSeq 6000 (~30,000 reads per cell). All scRNA-seq, library preparation, and sequencing were performed at the Baylor College of Medicine Single Cell Genome Sequencing Center. To analyze scRNAseq data, raw FASTQ files were demultiplexed and aligned to mouse reference genome (mm10) using Cell Ranger v7.1.0. Count files from Cell Ranger were imported into Seurat for quality control and subsequent analyses (4, 5). Briefly, cells were filtered by >200 unique genes, <10,000 number of RNA molecules, <30% mitochondrial genes, <3% Malat1 expression, and <0.01% Hba expression. Seurat objects were merged, normalized, and integrated using FindIntegrationAnchors. Cell clusters were identified by dimensionality reduction with principal component analysis (RunPCA) and shared nearest neighbor (SNN) analysis and visualized on UMAP plots. Differentially regulated cell-cell communication pathways were analyzed using CellChat v1.6.1 (6).

Western blotting: Immediately following PES on postoperative day three, mouse whole atrial tissue was snap-frozen in liquid nitrogen. 70 µL of RIPA lysis buffer containing 1% CHAPS, Phos-STOP (4906837001, Sigma-Aldrich, St. Louis, MO) and complete mini protease inhibitor cocktail (4693124001, Sigma-Aldrich, St. Louis, MO), 20mM sodium fluoride (NaF), 1mM Na_3VO_4 was added, and atrial tissue was homogenized with steel beads using a homogenizer (Tissue Lyser LT, Qiagen, Germantown, MD) at 50-Hz for 5 minutes. Samples were sonicated and centrifuged at 14,000 rpm for 20 mins at 4ºC. Supernatants were collected as protein lysates, and protein concentration was measured using a NanoDrop spectrophotometer (Thermo Fisher Scientific, Waltham, MA). 60 µg protein was loaded into each well of a 10-12% polyacrylamide gel run at 100 volts. Proteins were transferred onto a 0.45-µm polyvinylidene fluoride (PVDF) membrane for 1.5-h at 100 Volts at room temperature in ice cold Tris-Glycine transfer buffer containing 20% methanol. Membranes were blocked for 1-h at room temperature (catalog #20-314, Genesee Scientific, El Cajon, CA) and incubated overnight at 4°C with primary antibodies (F4/80, Cell Signaling Technology, catalog #70076, clone D2S9R, 1:1000; GAPDH, Proteintech, catalog #60004, clone 1E6D9, 1:5000; CXCR2, Proteintech, catalog #19538, 1:1000) diluted in blocking buffer (catalog #20-314, Genesee Scientific, El Cajon, CA). Membranes were washed 3 times with TBST (0.1% tween-20) for 10 minutes each and incubated with secondary antibody (goat anti-mouse, Invitrogen #A32729, 1:10,000; goat anti-rabbit, Invitrogen #A32735, 1:10,000) for 1-h at room temperature. After washing, membranes were developed using LICOR Odyssey infrared imager (LICOR, Lincoln, NE). Bands were quantified using ImageJ and normalized to GAPDH.

Flow cytometry: Flow cytometry of mouse atrial non-myocytes was done as previously described in detail (7). Briefly, atrial non-myocytes were isolated after terminal PES studies by mechanical and enzymatic digestion via Collagenase IV (320 U/mL; C4-22, Sigma-Aldrich, St. Louis, MO), Dispase II (1 U/mL; D4693, Sigma-Aldrich, St. Louis, MO), and DNase (60 U/mL; D45131VL, Sigma-Aldrich, St. Louis, MO) dissolved in phosphate-buffered saline (PBS) supplemented with Ca^{2+} (0.9 mM) and Mg^{2+} (0.5 mM) at 37ºC. Quenching was achieved with a solution of 60 U/mL DNase and 2% fetal bovine serum (FBS; TMS-016, Sigma-Aldrich, St. Louis, MO) in PBS without Ca^{2+} and Mg^{2+}. Cells were filtered through a 70-μm filter and spun at 340 x g for 7 minutes at 4°C. Red blood lysis was performed (catalog #00-4300-54, ThermoFisher Scientific, Waltham, MA) prior to live/dead staining (catalog #L34959; ThermoFisher Scientific, Waltham, MA) at room temperature for 15 minutes. Cells were resuspended in flow cytometry buffer (0.1% gelatin, 0.05% NaN_3 in PBS without Ca^{2+}/Mg^{2+}) and incubated in Fc block (0.01 μg/μL; catalog #553142, BD Biosciences, Franklin Lakes, NJ) for 5 minutes on ice. The following flow antibodies were used: CD45-PE-Cy5 (0.06 μg/test; catalog #15-0451-81, clone 30-F11, ThermoFisher Scientific, Waltham, MA), CD11b-PE (0.125 μg/test; catalog #12-0112-81, clone M1/70, ThermoFisher Scientific, Waltham, MA), and Ly6G-FITC (0.5 μg/test; catalog #11-9668-82, clone 1A8-Ly6g, ThermoFisher Scientific, Waltham, MA). Antibodies were added to the cell suspension and incubated at 4°C on a shaker for 45 minutes prior to imaging on a BD FACS Symphony. Data were analyzed using FlowJo v10.10.0. Debris was gated out using forward and side scatter area, followed by gating for singlets using forward scatter area and height and live cells using the live/dead stain.

Compensation was applied using singly-stained controls, and proper gating was determined using fluorescence minus one (FMO) controls.

Human Pericardial Fluid Samples: Pericardial fluid was collected from patients with pericardial drains placed for medical indications 24-36 hours after cardiac surgery. Experimental protocols were approved by the local institutional review board (#H-46755). Patients with prevalent infection were excluded. Pericardial fluid was spun for 5 minutes at 800 x *g* at 4C. The supernatant (i.e., cell-free fraction) was used to assess MIF protein levels by enzyme-linked immunosorbent assay (Proteintech #KE00248).

THP-1 cells: THP-1 cells were acquired as a generous gift from Dr. Huaizhu Wu (Baylor College of Medicine, Houston, Texas). Cells were maintained in suspension in RPMI 1640 media supplemented with Penicillin/Streptomycin, 1M HEPES, 100 mM sodium pyruvate, 250 g/L D-glucose, and 0.05 nM beta-mercaptoethanol. Following treatment with pericardial fluid or vehicle for 6-h, THP-1 monocyte suspension was spun at 500 x g for 5 minutes. For RNA isolation, the cell pellet was suspended in Trizol (15596, Life technologies, Carlsbad, CA), and 500 µg of RNA was reverse transcribed by iScript (1708841, Bio-Rad, Herclues, CA). The iTaq Universal SYBR Green Supermix (Fisher Scientific, Waltham, MA) with 1 µM primer and cDNA (1:25 dilution) were used for real-time quantitative polymerase chain reaction (RT-qPCR). The following RT-qPCR primers were used: *GAPDH* (Forward: CCACTCCTCCACCTTTGAC, Reverse: ACCCTGTTGCTGTAGCCA) and *IL1B* (Forward: AGCTGATGGCCCTAAACAGA, Reverse: TCGGAGATTCGTAGCTGGAT). The ΔΔCT method was used to calculate relative gene expression normalized to GAPDH. For protein isolation blotting, pelleted

cells were suspended in RIPA lysis buffer, followed by western blotting (see above). The following primary antibodies were used: phospho-Tyr705-STAT3 (Cell Signaling Technology #9145, 1:1000), total STAT3 (Cell Signaling Technology #9139, 1:1000), and GAPDH (Proteintech #60004, 1:5000).

Statistics: Statistics were performed using Prism version 10.1.1 (GraphPad, La Jolla, CA). All data points represent individual biological replicates unless otherwise stated. Continuous data are expressed as mean ± standard error of the mean (SEM). The D'Agostino-Pearson normality test was used to confirm normality. Two-sample t-tests or one-way ANOVA were used for parametric continuous data, and Mann-Whitney test or Kruskal-Wallis tests were used for non-parametric data. All two-sample t-tests were two-tailed. Categorical variables were evaluated with Fisher's exact or chi-square tests. Fisher's exact tests were used when expected counts were less than 5 in at least 80% of groups. For multiple group comparison, 1-way ANOVA or Kruskal-Wallis followed by Tukey or Dunn's post-hoc tests, respectively, were used to adjust for multiple testing with alpha=0.05. $P<0.05$ was considered statistically significant. Outliers were calculated by the ROUT method with a false discovery rate of 1% (8). Power analyses were conducted pre-hoc to determine sufficient sample size to detect statistically significant differences at $\alpha=0.05$ with 80% power.

REFERENCES

1. Keefe JA, Navarro-Garcia JA, Ni L, Reilly S, Dobrev D, and Wehrens XHT. In-depth characterization of a mouse model of postoperative atrial fibrillation. *J Cardiovasc Aging.* 2022;2.
2. Winner M, Meier J, Zierow S, Rendon BE, Crichlow GV, Riggs R, et al. A novel, macrophage migration inhibitory factor suicide substrate inhibits motility and growth of lung cancer cells. *Cancer Res.* 2008;68(18):7253-7.
3. Li N, and Wehrens XH. Programmed electrical stimulation in mice. *J Vis Exp.* 2010(39):e1730.
4. Luo W, Wang Y, Zhang L, Ren P, Zhang C, Li Y, et al. Critical Role of Cytosolic DNA and Its Sensing Adaptor STING in Aortic Degeneration, Dissection, and Rupture. *Circulation.* 2020;141(1):42-66.
5. Li Y, Ren P, Dawson A, Vasquez HG, Ageedi W, Zhang C, et al. Single-Cell Transcriptome Analysis Reveals Dynamic Cell Populations and Differential Gene Expression Patterns in Control and Aneurysmal Human Aortic Tissue. *Circulation.* 2020;142(14):1374-88.
6. Jin S, Guerrero-Juarez CF, Zhang L, Chang I, Ramos R, Kuan CH, et al. Inference and analysis of cell-cell communication using CellChat. *Nat Commun.* 2021;12(1):1088.
7. Keefe JA, Aguilar-Sanchez Y, Navarro-Garcia JA, Ong I, Li L, Paasche A, et al. Macrophage-mediated interleukin-6 signaling drives ryanodine receptor-2 calcium leak in postoperative atrial fibrillation. *J Clin Invest.* 2025.

8. Motulsky HJ, and Brown RE. Detecting outliers when fitting data with nonlinear regression - a new method based on robust nonlinear regression and the false discovery rate. *BMC Bioinformatics.* 2006;7:123.

SUPPLEMENTAL FIGURES

Figure S1. *Mif* is upregulated in *Acta2*⁺ AFBs in TAF versus sham mice. (**A**) Dot plot of top genes expressed by each AFB and macrophage cluster showing *Mif* to be enriched in *Acta2*$^{+}$ AFBs (FB4). (**B**) Cell-cell communication analyses using CellChat revealed MIF to be a top differentially upregulated outgoing signaling pathway mediated by FB4. (**C**) *Mif* expression was greater in AFBs and macrophages in TAF versus sham mice. Red arrow denotes FB4. *Abbreviations*: AFB - atrial fibroblast, ANGPTL - angiopoietin-like protein, COMP - complement, GAL - galectin, POSTN - periostin, SPP1 - secreted phospho-protein 1, TAF - thoracotomy atrial fibrillation.

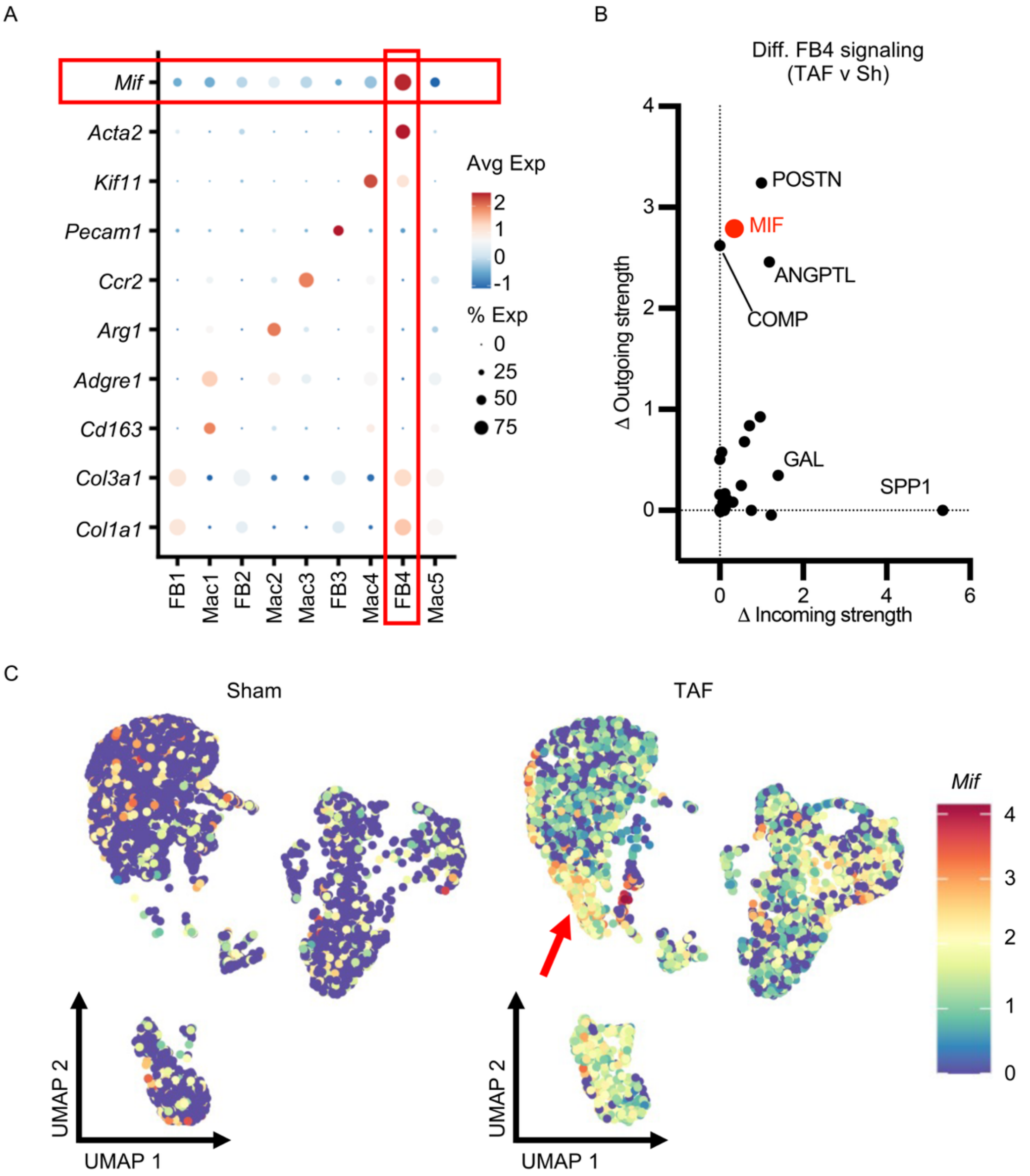

Figure S2. MIF inhibition attenuates poAF duration in mice. (**A**) Representative ECG traces after burst pacing in sham (top), thoracotomy mouse treated with vehicle (middle), and thoracotomy mouse treated with MIF inhibitor (bottom). (**B**) Quantification of poAF duration (Kruskal-Wallis). Each dot in (B) denotes one mouse (N=10 Sh, N=17 Th, N=9 MIF-i). *Abbreviations*: ECG - electrocardiogram, MIF-i - MIF inhibitor, Sh - sham, Th - thoracotomy, Veh - vehicle.

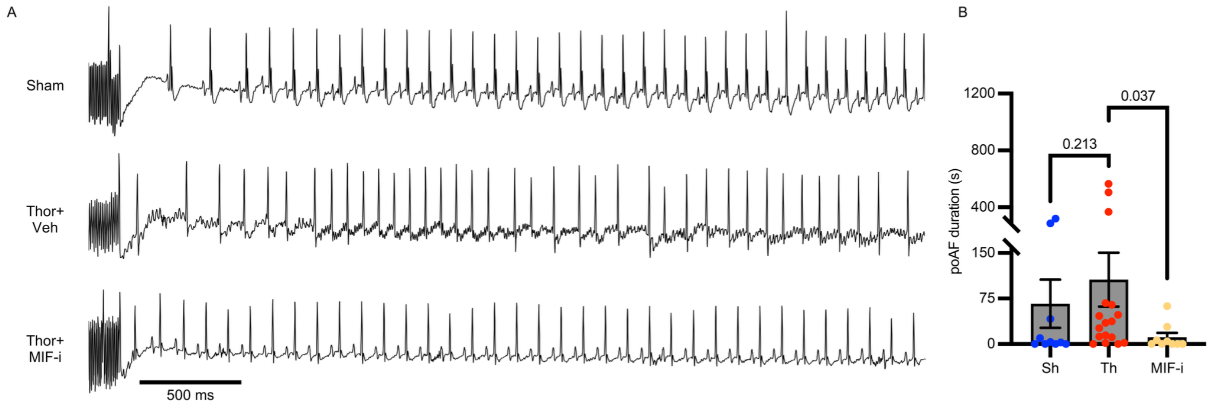
A
Sham
Thor+
Veh
Thor+
MIF-i
500 ms
B
1200
800
400
150
75
0
poAF duration (s)
0.213
0.037
Sh
Th
MIF-i

Figure S3. MIF inhibition does not alter atrial PMN infiltration after thoracotomy. MIF was inhibited by 4-IPP given at 40 mg/kg/day from POD 0-2, followed by flow cytometric staining of atrial non-myocytes for CD45, CD11b, and Ly6G. PMNs were defined as CD45$^+$/CD11b$^+$/Ly6G$^+$ cells. (**A-B**) TAF mice had trended toward greater atrial PMN accumulation compared to sham mice whereas no differences were seen after MIF inhibition. N=4 mice per group. *Abbreviations*: MIF - macrophage migration inhibitory factor, PMN - polymorphonuclear neutrophil, POD - postoperative day, Sh - sham, TAF - thoracotomy atrial fibrillation.

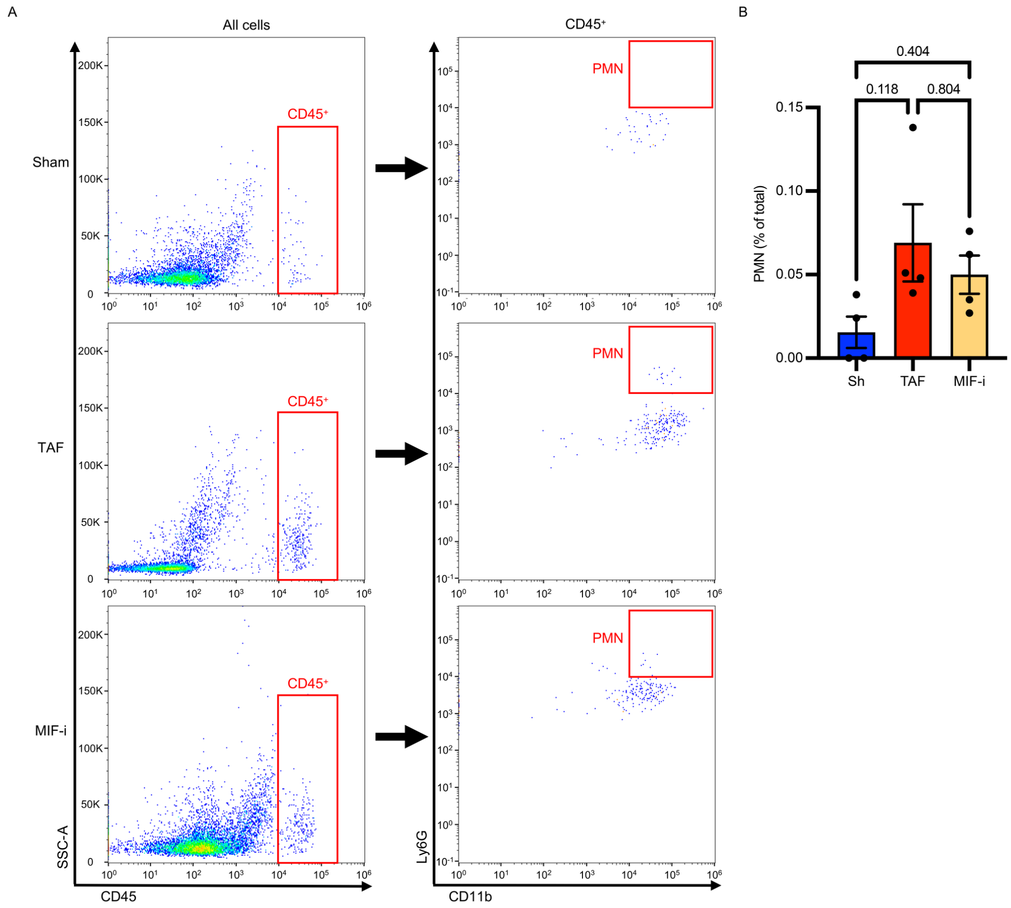

Figure S4. MIF inhibition prevents PF-driven *IL1b* upregulation in THP-1 monocytes. Undifferentiated THP-1 monocytes were treated for 6-h with vehicle (blue), PF from SR patients (green), PF from poAF patients (red), and PF from poAF patients after 30-minute pretreatment with MIF-inhibitor 4-IPP at 100 μM (yellow). RT-qPCR from treated THP-1 monocytes revealed significantly greater *IL1B* expression that was attenuated by MIF inhibition (1-way ANOVA). Each dot represents an individual well. N=4 per group. *Abbreviations*: IL1B - interleukin-1 beta, PF - pericardial fluid, poAF - postoperative atrial fibrillation, SR - sinus rhythm, Veh - vehicle.

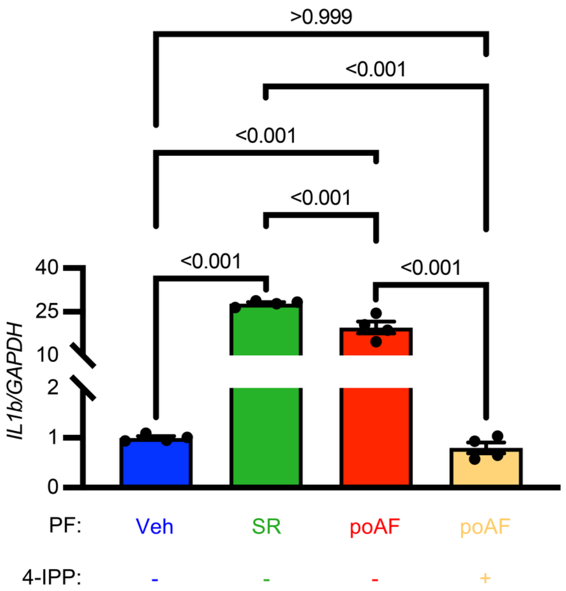

SUPPLEMENTAL TABLES

Table S1. Demographic and clinical characteristics of cardiac surgery patients.

	**SR**	**poAF**	***P* value**	**Test**
Patients, n	6	11	N/A	N/A
Female/Male	4/2	4/7	0.335	Fisher's
Age, y (mean±SD)	53.3 ± 11.7	63.1 ± 7.6	0.106	T-test
CABG, n (%)	4 (67)	5 (45)	0.620	Fisher's
Aortic surgery, n (%)	1 (17)	3 (27)	>0.999	Fisher's
Valvular surgery, n (%)	1 (17)	2 (18)	>0.999	Fisher's
CABG + valvular, n (%)	0 (0)	1 (9)	>0.999	Fisher's
Beta blockers, n (%)	5 (83)	7 (64)	0.600	Fisher's
Lipid lowering, n (%)	4 (67)	7 (64)	>0.999	Fisher's
Aspirin, n (%)	4 (67)	3 (27)	0.162	Fisher's
DHP CCBs, n (%)	3 (50)	4 (36)	0.644	Fisher's
ACEi/ARB, n (%)	3 (50)	6 (55)	>0.999	Fisher's
Diuretics, n (%)	3 (50)	5 (45)	>0.999	Fisher's
Hypertension, n (%)	5 (83)	9 (82)	>0.999	Fisher's
Hyperlipidemia, n (%)	6 (100)	6 (55)	0.102	Fisher's
Diabetes mellitus, n (%)	4 (67)	6 (55)	>0.999	Fisher's
CAD, n (%)	5 (83)	6 (55)	0.333	Fisher's
Valvular disease, n (%)	2 (33)	3 (27)	>0.999	Fisher's

Abbreviations: ACEi - angiotensin converting enzyme inhibitor, ARB - angiotensin receptor blocker, CABG - coronary artery bypass grafting, CAD - coronary artery disease, CCBs - calcium channel blockers, DHP - dihydropyridine.
