# Supplementary material for: Atrial fibroblast–derived macrophage migration inhibitory factor promotes atrial macrophage accumulation in postoperative atrial fibrillation
Source: JCI Insight. 2025 Aug 14;10(18):e190756. doi: 10.1172/jci.insight.190756 (PMC12487863; doi:10.1172/jci.insight.190756)

Unedited gels

→ **denotes band of interest**

☐ **denotes representative blot used in figure**

L **denote ladder**

X **denotes samples that were excluded**

Full, unedited blots for Figure 1F – gel 1

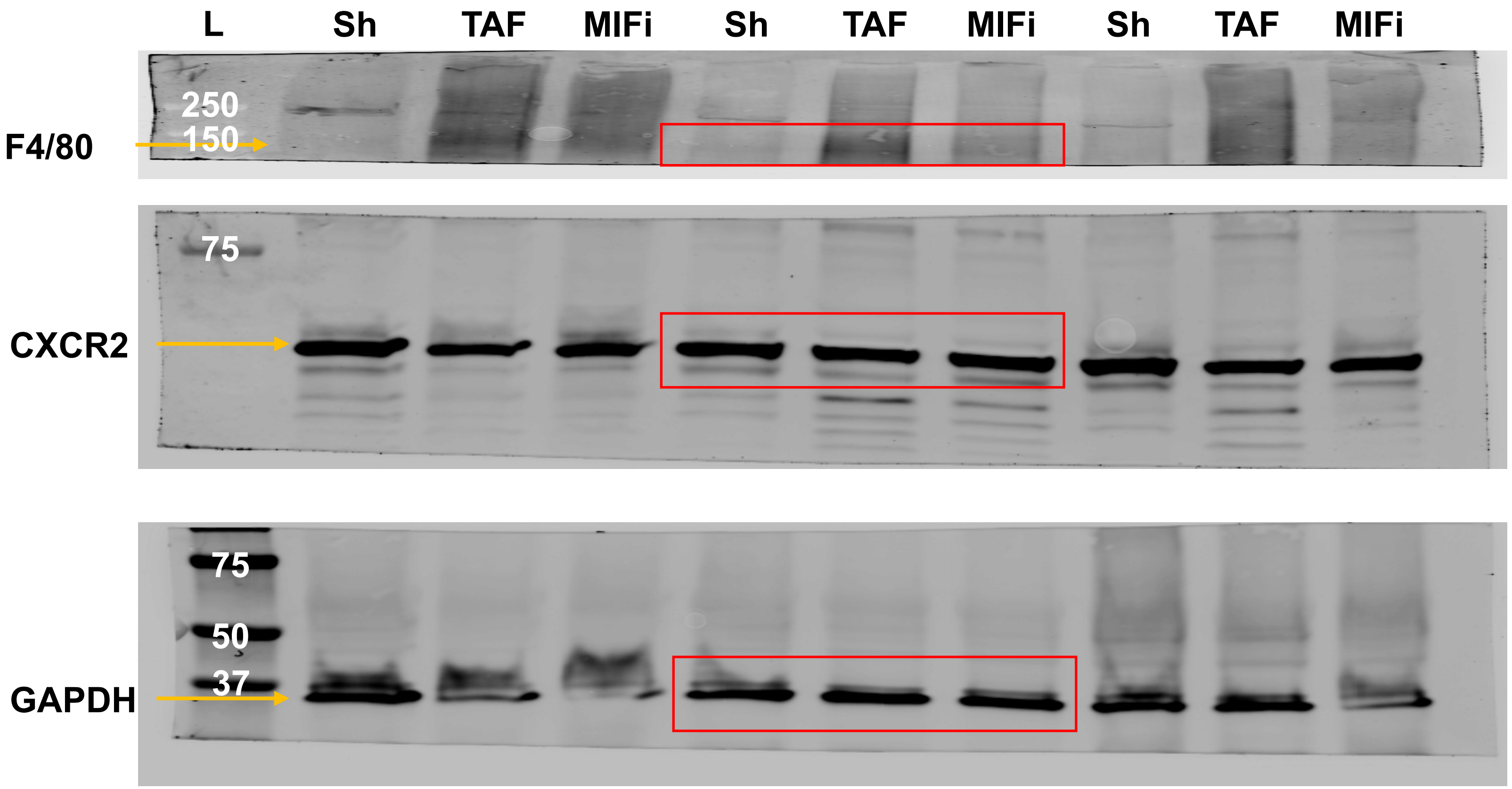

Full, unedited blots for Figure 1F – gel 2

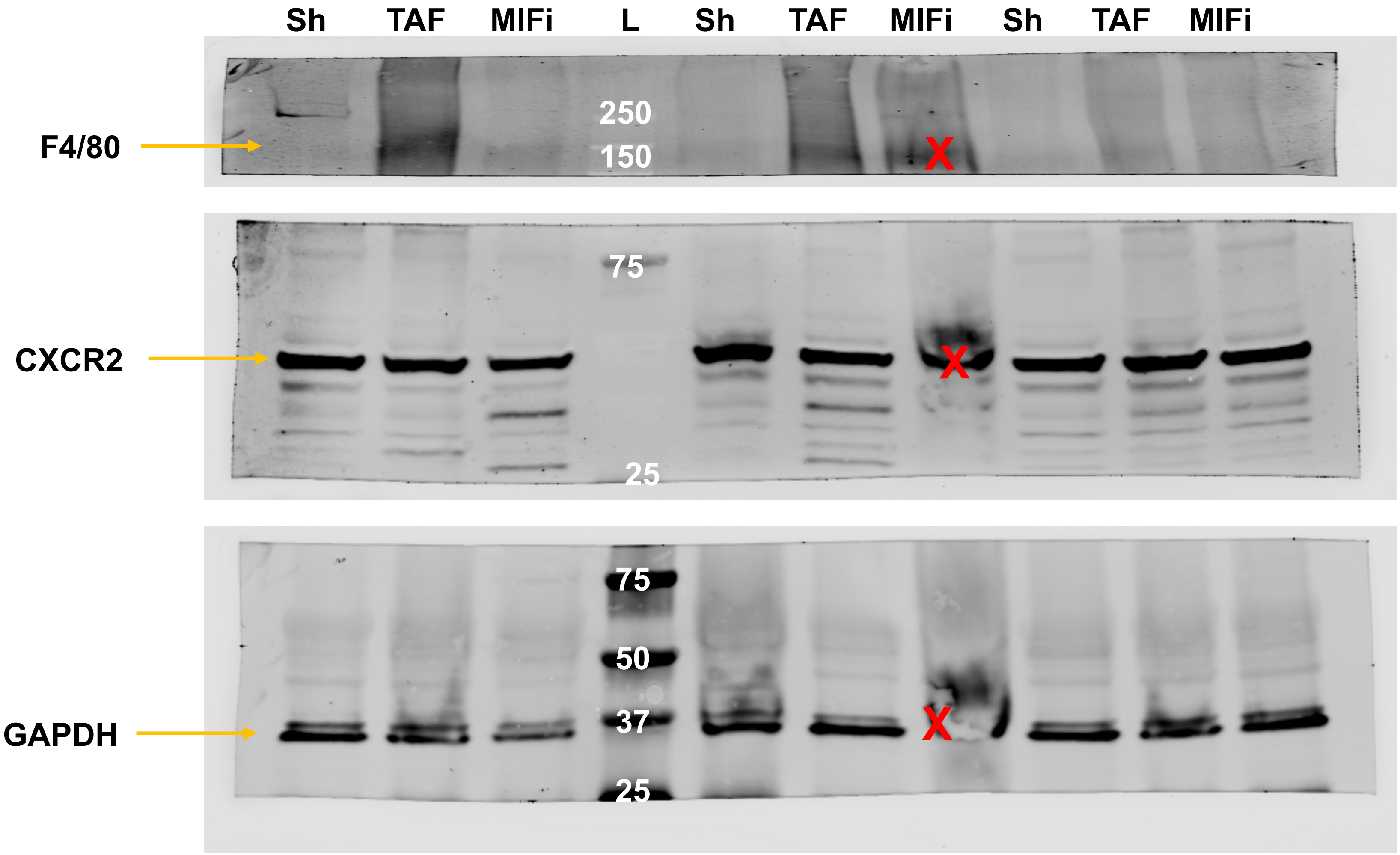

Full, unedited blots for Figure 1I – gel 1

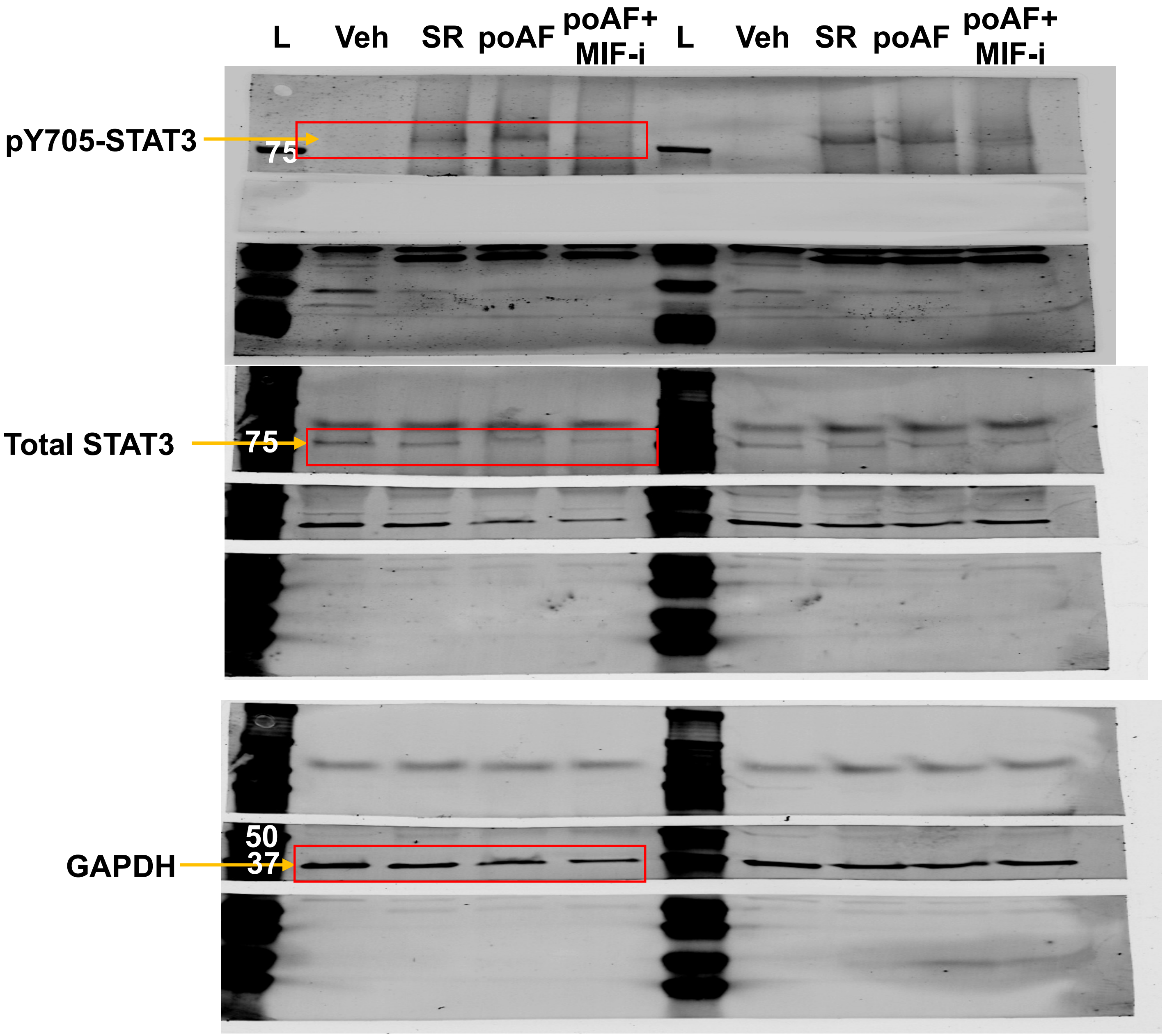

Full, unedited blots for Figure 1I – gel 2

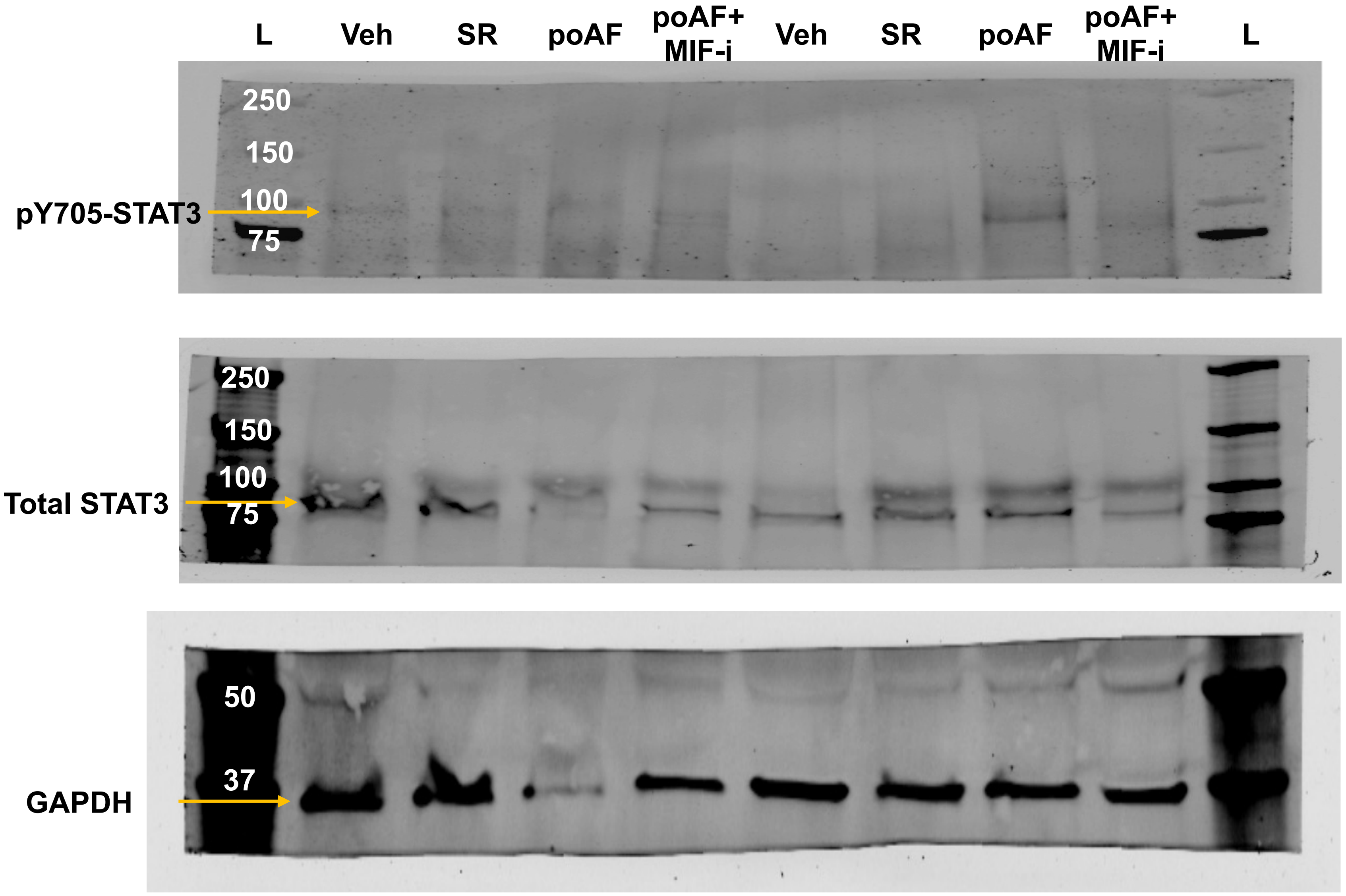

Supplement: Unedited blot and gel images [file jciinsight-10-190756-s064.pdf]
